# Comment on “Trichobezoar Causing Airway Compromise during Esophagogastroduodenoscopy”

**DOI:** 10.1155/2015/450467

**Published:** 2015-10-26

**Authors:** Antonios Athanasiou, Demetrios Moris, Eleftherios Spartalis

**Affiliations:** 1st Surgery Department, Laikon General Hospital, National and Kapodistrian University of Athens, Agiou Thoma Street 17, Attiki, 11527 Athens, Greece

We read with great interest the article by Kao et al. [1] published in the September 2015 issue of Case Reports in Medicine, which highlighted how important is the careful attention to airway management during esophagogastroduodenoscopy for removal of a trichobezoar and similar foreign body. However, until today, it is still controversial which is the best option for the treatment of gastric bezoar. Endoscopic therapy can be efficient for bezoars composed of milk curd (lactobezoars) and vegetable matter (phytobezoars) because usually they are small in size, but it is difficult to be efficient for trichobezoars, especially those that are large (>20 cm) [2]. Several factors should be taken into consideration before the final decision, such as the presence of complications and the trichobezoar's consistency as well as the size and localization. The complications during the esophagogastroduodenoscopy for removal of a trichobezoar are estimated which are not uncommon but just unreported. Park et al. [3] have reported a 12.8% morbidity rate for endoscopic treatments of 39 patients. In another earlier study, Erzurumlu et al. [4] reported a 14% endoscopic morbidity rate, while Spyridon et al. [5] have reported only 11% morbidity rate. The most common complications that have been reported after treatment are bleeding, obstruction, ileus, fever, and perforation. Last but not least, piecemeal removal of large size lesions should be avoided because secondary bezoars are possible to migrate leading to more complications.

According to our experience with similar cases which were treated by surgical treatment, the combination of trichobezoars and gastric ulcers is very common [6]. In this case report, the authors do not mention if they repeat gastroscopy after the extraction of the trichobezoar, or if they intend to do so at the follow-up. Many cases related to trichobezoars are presented to the emergency department with symptoms of perforative acute abdomen and epigastric mass [7]. According to the literature, the presence of gastric ulcers simultaneously with trichobezoars is not an uncommon combination, which means that it is necessary for the surgeon to inspect carefully for peptic ulceration after the extraction of the trichobezoar. While it is certainly true that endoscopic approach has excellent results for small trichobezoars (i.e., <6 cm), for larger masses, surgical approach is simple, with high success rate and low complication rate. Furthermore, elective laparotomy gives to the surgeon the chance to examine carefully the total gastrointestinal track for possible satellites.
